# High Functional Stability of a Low-cost HBV DNA qPCR Primer Pair and Plasmid Standard

**DOI:** 10.5005/jp-journals-10018-1160

**Published:** 2016-07-09

**Authors:** Jorge Aguiar, Gerardo García, Yamila León, Eduardo Canales, José Angel Silva, Omar Gell, Regla Estrada, Ivis Morán, Verena Muzio, Gerardo Guillén, Eduardo Pentón, Julio Cesar Aguilar

**Affiliations:** 1 Department of Therapeutic Vaccine against Hepatitis B, Center for Genetic Engineering and Biotechnology, Havana, Cuba; 2 Department of Quality Control, Center for Genetic Engineering and Biotechnology, Havana, Cuba; 3 Department of Plant Genomics, Center for Genetic Engineering and Biotechnology, Havana, Cuba; 4 Department of Oligonucleotide Synthesis, Center for Genetic Engineering and Biotechnology, Havana, Cuba; 5 Department of Plant Environmental Biotechnology, Center for Genetic Engineering and Biotechnology, Havana, Cuba; 6 Department of Clinical Studies, Center for Genetic Engineering and Biotechnology, Havana, Cuba

**Keywords:** Cycles threshold, Functional stability, Hepatitis B virus, Laboratory research, Oligonucleotide, Plasmid, Primer, Quantitative PCR.

## Abstract

**Aim:**

We studied the functional stability of a primer pair and the standard curve based on a plasmid carrying full-length HBV genome, from a novel low-cost real-time quantitative polymerase chain reaction (qPCR) assay. The assay was developed at the Center for Genetic Engineering and Biotechnology (CIGB) in Havana, to quantify the serum hepatitis B virus (HBV) DNA from chronic HBV-infected (CHB) patients.

**Materials and methods:**

In-house generated oligonucleotides and plasmids were incubated at 37°C during 1 month and compared with the same materials incubated at –20, 4, and 25°C during the same time in qPCR experiments.

**Results:**

This work shows that the oligonucleotide pair and the plasmid for the quantitative standard curve are functionally stable in severe temperature conditions during 1 month. Polymerase chain reaction amplification with both materials after its incubation 30 days at 37°C produced similar cycle threshold (CT) values and similar degree of sample quantifications compared with the same materials preserved using the conventional storage conditions at –20°C.

**Conclusion:**

These results are indicative of the robustness of this low-cost qPCR system for HBV DNA quantification. These results also support that this qPCR assay can be used as a low-cost technology in clinical studies to monitor the viral load changes of serum HBV DNA of CHB patients, which could be used by poor people of third world countries, where there are frequent blackouts and temperature changes that can hinder the primer and plasmid stability.

**How to cite this article:**

Aguiar J, García G, León Y, Canales E, Silva JA, Gell O, Estrada R, Morán I, Muzio V, Guillén G, Pentón E, Aguilar JC. High Functional Stability of a Low-cost HBV DNA qPCR Primer Pair and Plasmid Standard. Euroasian J Hepato-Gastroenterol 2016;6(1):19-24.

## INTRODUCTION

Hepatitis B virus (HBV) is the most common cause of chronic viral hepatitis in human beings worldwide.^1^ Over 400 million HBV-infected patients are chronic virus carriers, and over 20% of them will develop complications, such as liver cirrhosis and hepatocellular carcinoma.^1^

The quantitative determination of the HBV level in blood (viral load) is a basic variable for the follow-up and classification of the condition of chronic hepatitis B (CHB) patients. Currently, viral load (VL) is the main variable used to measure the efficacy of therapeutic products for this disease.^2-4^ Common commercial systems for HBV DNA quantification are very expensive for patients of developing and resource-constrained countries. Therefore, it is essential to develop a quantitative method to detect the blood VL equivalent to the current international standards and thus minimize expenses for the health systems and patients.^3-5^

Last year we described the characteristics and validation experiments of a cheap and simple quantitative real-time PCR (qPCR) method^6^ that uses an unspecific and not very expensive commercial amplification kit (the Quantitect SYBR Green PCR kit, Qiagen, Germany). This was combined with the standards (based on a plasmid carrying the full-length HBV genome) and specific primers for the S gene, both produced at the Center for Genetic Engineering and Biotechnology (CIGB). The new combined kit was used to measure the concentration of HBV DNA in serum samples of Cuban HBV carriers. The new low-cost qPCR combined system was compared with other commercial qPCR systems such as “artus HBV LC PCR kit” (Qiagen) (R = 0.90) and “HBV Monitor system” (Roche) (R = 0.82). This demonstrated that the new system performed at the same level of quality than two of the most common international systems used for HBV DNA quantification.^6^

In the present paper, we demonstrate that the oligonucleotide pair and the plasmid used for the standard curve in the new qPCR system for HBV DNA as reported by Aguiar et al 2014^6^ are functionally very stable in severe working conditions (1 month incubation at 37°C). The results supported the robustness of the primer pair and the standard curve based on a plasmid carrying the complete HBV genome of 3.2 kb length from the above-mentioned qPCR system for HBV VL quantification.^6^

## MATERIALS AND METHODS

### Subjects

Serum HBV DNA from two CHB patients was used as positive control samples for the stability assay: CC6 with VL of 5.3 × 10^6^ copies/mL (26,500 copies/reaction) and CC8 with VL of 2.4 × 10^6^ copies/mL (12,000 copies/reaction). Both patients previously diagnosed by Aguiar et al 2014^6^ were recruited from the “Camilo Cienfuegos” Hospital in Sancti-Spiritus, in the Cuban central region.

### HBV DNA Quantification

*HBV DNA purification:* The HBV DNA of the two CHB patients was purified from 200 µL of serum with the “QIAamp DNA Mini kit” (Qiagen, Germany), according to the manufacturer’s instructions.

*Quantitative PCR reaction:* qPCR reaction was prepared as described previously by Aguiar et al^6^ using the unspecific commercial SYBR Green reaction mix from the “Quantitect SYBR Green PCR kit” (Qiagen, Germany).

*Primers:* The oligonucleotide pair used at a concentration of 0.67 µM was previously published by other authors.^7,8^ The sequences of both are: sense 5′-GTGTCTGCGGCGTTTTATCA-3′ and antisense 5′- ACAAACGGGCAACATACCTT-3′.

*Thermal cycling:* Thermal cycling was performed in a Rotor Gene 3000 Real-time PCR (Corbett Research, Australia). Reaction conditions were 95°C for 15 minutes followed by 40 cycles of 94°C for 15 seconds, 58°C for 30 seconds, and 72°C for 30 seconds.

*Standard curve:* The pST012012 plasmid (pST012012. Plasmid bank of CIGB, 2012) that carries the complete 3.2 kb genome of the HBV was used as the quantification standard curve in the qPCR of the functional stability assay, as previously described by Aguiar et al.^6^ We usually use a five-point standard curve: 9 × 10^6^ c/r (1.8 × 10^9^ copies/mL), 9 × 10^4^ c/r (1.8 × 10^7^ copies/mL), 9 × 10^2^ c/r (1.8 × 10^5^ copies/mL), 9 c/r (1.8 × 10^3^ copies/mL), and 0.09 c/r (18 copies/mL). qPCR positive controls (C+): Each qPCR test run included a positive control (C+) sample (CHB patient CC6 or CC8).

### Description of the Experiments of Stability in Extreme Conditions for the Primer Pair and for the Standard Curve Points Based on the pST012012 Plasmid

The extreme condition employed to know the stability of the primer pair and the five standard curve points based on the pST012012 plasmid was the incubation of these two materials at 37°C during 1 month. The concentration employed of both materials in the stability experiments were the “work concentration” in qPCR: Primers (10 pmol/µL) and plasmid (as we described for the five standard curve points in the “Materials and Methods” section). Other temperatures also were proved to compare with the extreme condition during the same 1 month of incubation time: –20°C (positive control of normal DNA storage), 4°C and 25°C.

### Statistical Analysis

The variables followed to study the stability of plasmid and oligonucleotides were the CT values and the estimated concentration of the positive controls. These parameters were analyzed by regression using the temperature as independent variable.

In the case of CT values behavior in function of the temperature, all points of standard curve and positive control were taken into account, but for the estimated concentration, since it is determined using the plasmid curve as the reference, just the controls were analyzed.

First, an independent regression of each point of the standard curve and positive controls (for CT) or just positive controls (for the estimation of the concentration) was carried out, and the difference between the slopes was investigated. If the difference was not significant, a common slope regression was carried out to increment the degrees of freedom of the residual variance and hence the statistical power used to determine if there is significant variation of threshold or estimated concentration with temperature. The significance level for the equality of slopes was 0.25, while for the dependence of parameters with temperature, it was 0.05.

## RESULTS

We developed the qPCR experiments for the study of the functional stability after incubation at 37°C during 1 month of the two primers and the five standard curve points employed in a low-cost qPCR system to quantify HBV, both materials diluted at the work concentration, as described by Aguiar et al.^6^ Tables 1 and 2 represent the results obtained in the qPCR to evaluate the stability of the primer pair by means of the CT (Table 1) and estimated concentration of the hepatitis B VL from a positive control serum sample, expressed in copies/reaction (c/r) (Table 2).

**Table 1:** **Table 1:** Analysis of quantitative polymerase chain reaction experiment about the differences among the primer pair when they were incubated at different temperatures employed in the stability assay, in function of the cycle threshold

						*CT (in qPCR for the five standard curve points and C+)*													
*Temp (°C)*		*Time (days)*				*900,000*		*9,000*		*90*		*0.9*		*0.09*		*C+1*		*C+2*	
37		30		Primer pair		16.86		21.23		29.86		36.53		38.43		21.52		21.46	
25		30		Primer pair		13.43		17.76		25.47		30.28		32.44		17.44		17.47	
4		30		Primer pair		13.72		17.49		25.44		31.79		31.87		18.07		17.45	
–20		30		Primer pair		13.63		17.72		25.07		31.84		34.86		17.35		16.96	

Standard curve points (all stored at –20°C during this experiment)

**Table 2:** **Table 2:** Viral load differences in qPCR among the primer pair when they were incubated at the different temperatures employed in the stability assay, in function of the quantification of the positive control (C+) serum sample CC6 by duplicate expressed in copies/reaction (c/r)

						*VL quantification expressed in c/r for the positive control (C+)*					
*Temp (°C)*		*Time (days)*				*C+1*		*C+2*	
37		30		Primer pair		20,735.65		21,679.36	
25		30		Primer pair		27,345.92		26,676.60	
4		30		Primer pair		18,177.53		29,944.33	
–20		30		Primer pair		29,712.75		39,602.49	

Standard curve points (all stored at –20°C during this experiment)

The results of the qPCR experiments illustrated in Table 1 refer only to the analysis of the differences among the primer pair (after the incubation at the different temperatures: 37°C, 25°C, 4°C, and –20°C), in function of the CT obtained in the quantification of the hepatitis B VL from the five standard curve points and a positive control (C+) serum sample by duplicate (CC6).

Table 2 illustrates the results of the qPCR experiments that refer only to the analysis of the differences among the primer pair (after the incubation at the different temperatures: 37°C, 25°C, 4°C, and –20°C), in function of the quantification of the hepatitis B VL expressed in c/r for the C+ serum sample (CC6) by duplicate.

**Graphs 1A and B:**
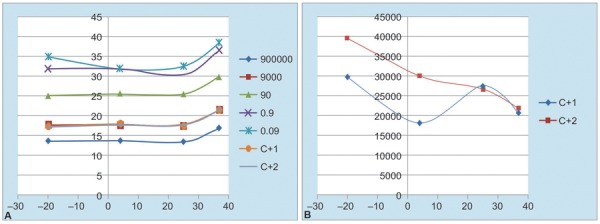
Variation of the cycle threshold after 1 month incubation of the primer pair at 37°C, 25°C, 4°, and –20°C: (A) Variation of the CT in qPCR from the five plasmid standard curve points and the positive control (C+) CC6 by duplicate after 1 month incubation of the primer pair at different temperatures, (B) Variation of the estimated concentration expressed in copies/reaction (c/r) in qPCR of the positive control (C+) CC6 by duplicate after 1 month incubation of the primer pair at different temperatures

Graphs 1A and B show the behavior of the CT and estimated concentration in function of the incubation temperature applied to the primer pair.

As can be seen from Graph 1A, the CT shows only a little variation with incubation temperature of the oligonucleotides. This variation can be seen after exposition of the primers at 37°C. Regression analysis shows significant variation of CT with temperature (p = 0.013) but only of 0.04 cycles/°C.

Estimated concentration shown in Graph 1B have no systematic variation with the incubation temperatures of the oligonucleotide pair, and only reflect the normal variability of the technique. In this case no significant dependence of concentration with the studied temperatures was found (p = 0.056).

A similar set of stability assays were realized also to the standard curve points based on the pST012012 plasmid that is employed in the low-cost qPCR for hepatitis B VL quantification system reported by Aguiar et al.^6^ In the next two sets of experiments that we will describe in the following issue, we employed only four of the five standard curve points that we typically used (as described in the “Materials and Methods” section).

Tables 3 and 4 represent the results obtained in the qPCR to evaluate the stability of only four standard curve points in function of the CT (Table 3) and in function of the quantification of a C+ serum sample, expressed in c/r (Table 4).

**Table 3:** **Table 3:** Analysis of qPCR experiment about the differences among the four standard curve points when they were incubated at the different temperatures employed in the stability assay, in function of the cycle threshold

						*CT (in qPCR for the four standard curve points and C+)*											
*Temp (°C)*		*Time (days)*				*9,000*		*90*		*0.9*		*0.09*		*C+1*		*C+2*	
37		30		Standard curve points		19.58		26.14		32.62		34.84		19.51		19.64	
25		30		Standard curve points		19.82		26.51		32.41		36.42		19.35		19.27	
4		30		Standard curve points		18.81		26.61		33.22		35.64		18.98		19.41	
–20		30		Standard curve points		19.35		26.07		31.44		34.65		18.90		19.54	

Primer pair always stored at –20°C during this experiment

**Table 4:** **Table 4:** Viral load differences in qPCR among the four standard curve points when they were incubated at the different temperatures used in the stability assay, in function of the quantification of the positive control (C+) sample CC8 by duplicate expressed in copies/reaction (c/r)

				*VL quantification expressed in c/r of the positive control (C+)*									
*Temp (°C)*		*Time (days)*				*C+1*		*C+2*	
37		30		Standard curve points		10, 915.15		9,947.29	
25		30		Standard curve points		12,573.01		13,335.12	
4		30		Standard curve points		10,709.58		8,026.58	
–20		30		Standard curve points		15,072.34		9,285.06	

Primer pair always stored at –20°C during this experiment

The results of the qPCR experiments illustrated in Table 3 refer only to the analysis of the differences among the four standard curve points (after the incubation at the different temperatures: 37°C, 25°C, 4°C, and –20°C), by mean of the CT obtained in the quantification of the hepatitis B VL from these proper four standard curve points and the C+ serum samples (CC8) by duplicate.

Table 4 illustrates the results of the qPCR experiments that refer only to the analysis of the differences among these four standard curve points (after the incubation at the different temperatures: 37°C, 25°C, 4°C, and –20°C), in function of the quantification of the hepatitis B VL expressed in c/r for the C+ serum sample (CC8) by duplicate.

**Graph 2:**
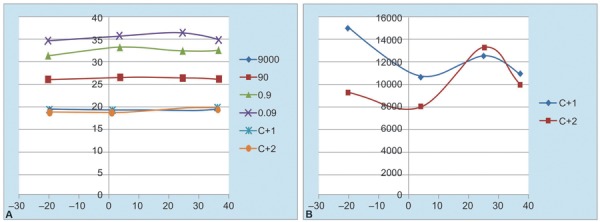
Graphs 2A and B: Variation of the cycle threshold after 1 month incubation of the plasmid standard curve points at 37°C, 25°C, 4°C, and –20°C: (A) Variation of the CT in qPCR from the four plasmid standard curve points and the positive control (C+) CC8 by duplicate after 1 month incubation of the plasmid standard curve points at different temperatures, and (B) Variation of the estimated concentration expressed in copies/reaction (c/r) in qPCR of the positive control (C+) CC8 by duplicate after 1 month incubation of the plasmid standard curve points at different temperatures

Graphs 2A and B shows the behavior of CT and estimated concentration in function of the incubation temperature applied to the standard curve points.

No variation of CT in function of incubation temperature of plasmid can be appreciated in Graph 2A. Regression analysis confirms this observation and shows no significance of CT in function of incubation temperature (p = 0.091).

Estimated concentration has not defined the tendency of variation with temperature. In Graph 2B just the variation proper of the technique can be appreciated. Statistical analysis shows no significance of estimated concentration with incubation temperature either (p = 0.803).

## DISCUSSION

As can be seen in Table 1 and Graph 1A the CT shows only a little variation with primer pair incubation temperature. CT values produced by the primers stored at –20°C or incubated for 1 month at 4°C and 25°C are the same, while CT values obtained with the primers incubated at 37°C are slightly higher. This increment in CT values represent a delay in the generation of qPCR products, which could mean that some damages in the primer’s DNA occur during the 1 month incubation at 37°C. This variation was proved to be significant, but it is small enough (0.04 cycles/°C) to produce only a 10% difference between results with the primers stored at –20°C and incubated at 37°C for 1 month, which is comparable with the day-to-day variation of the technique. Significance of CT in function of temperature was detected by statistical analysis, despite that it was small, due to high number of degrees of freedom in common slope regression, which confer a high statistical power to the test.

On the contrary, when we compare the values of the quantification of the C+ CC6 in Table 2 and Graph 1B, we can observe that the values of the quantification experiment from the –20°C control temperature and from rest of the temperatures studied are very similar among then. We can not observe evident differences among results produced by the four temperatures applied to the primer pair regarding the values of the relative quantification of hepatitis B VL (this observation was confirmed by statistical analysis as stated above). These results support the functional stability of the primer pair with respect to the relative quantification of the hepatitis B VL in a range of temperatures from –20°C to 37°C during 1 month of incubation time.

This behavior related to the similar quantification of the hepatitis B VL observed in qPCR for the C+ among the primer pair that were incubated previously at 37°C, 25°C, 4°C, and –20°C, although at 37°C the CT values were slightly superior when they were compared with the CT values obtained at the other temperatures studied, could be possible perhaps because there are still enough quantity of primer’s DNA that were not affected by the temperature of 37°C and still can be working correctly in the qPCR experiments. These results are in correspondence with the facts that the primer pair employed in the low-cost qPCR for hepatitis B VL quantification reported by Aguiar et al 2014^6^ can function correctly from a range of concentration of 0.23 to 0.67 mM.

The second set of experiments (Tables 3 and 4, and Graphs 2A and B) used only four of the usually five points of the standard curve constructed with pST012012 plasmid. In this case the plasmid instead of the primer pair was incubated at 37°C, 25°C, 4°C, and –20°C and we do not find any differences among CT values of the standard curve points or C+ obtained with the plasmid incubated at different temperatures. We neither found differences in the quantification of VL obtained in qPCR for the C+ CC8 with the four standard curves based on the pST012012 plasmid incubated at the above-mentioned temperatures. In both types of analysis (CT values and quantification of VL) statistical analysis confirmed the nonexistence of significant differences produced by incubation at the different temperatures assayed.

Quantitative real-time PCR (qPCR) has become a gold standard for the quantification of nucleic acids and microorganism abundances, in which plasmid DNA carrying the target genes are most commonly used as the standard due to its high stability (very little degradation during storage) and ease in preparation.^9,10^

Besides, plasmid supercoiled DNA that target heterologous genes from other organisms could be generating overestimations when they are used as a standard curve in qPCR.^10^ Nevertheless, the same authors explained that the only possible exception would be when the target DNA itself is circular (especially if it is in a supercoiled state), such as uncut mitochondrial, viral, bacterial, or plasmid DNA, in which whether the linear standard still gives more accurate result needs to be individually investigated.^10^

Naturally, the 3.2 kb genome of the HBV is organized in the form of covalently closed circular DNA (cccDNA) inside the nucleus of host cells or outside the nucleus in the form of capsid-associated relaxed circular DNA (rcDNA).^11,12^ Both forms of HBV DNA organization (rcDNA and cccDNA) can be coexisting in the liver and the sera of the infected patients.^13^ This fact motivated us to consider the strategy to employ as the standard curve for HBV DNA quantification the supercoiled form of the pST012012 plasmid that carries the full-length HBV genome,^6^ very similar to the natural target of HBV DNA that is present in the serum of the CHB patients (the HBV rcDNA and/or the cccDNA), in order to build a low-cost system based on qPCR that uses besides an unlabeled primer pair both materials generated in house. Our strategy included also the idea that both above-mentioned materials could have a high functional stability, as we found and reported in this study. Finally, we consider SYBR Green as the detector agent of the qPCR product. These three practical elements together constitute a novelty per se, directed to create an inexpensive qPCR system for HBV DNA quantification, which could be useful in countries and hospitals in the developing world with economic limitations, places where there are frequent power cuts, generating continuous temperature changes that could affect the primer and plasmid stabilities.

Although plasmid DNA carrying the target genes are the most commonly used as the standard curve in qPCR,^10^ there are not much publications of “in house systems” that use plasmid DNA carrying fragments of HBV genome in order to quantify amounts of HBV DNA in the human sera of the chronic patients, for example, the report of de Oliveira dos Santos et al 2014.^14^ To date, we have found no other publication in the literature like the qPCR system reported by Aguiar et al 2014^6^ that uses the supercoiled form of a plasmid carrying the full-length HBV genome as a standard curve for HBV DNA quantification. This system for quantification of the HBV DNA from sera of chronic patients is not only a simple low-cost system but also a sensible and accurate methodology comparable to other specific commercial kits available now in the marker.

## CONCLUSION

We can conclude that both the primer pair and the standard curve based on a plasmid employed in the qPCR system for the hepatitis B VL quantification published previously by Aguiar et al 2014,^6^ are functionally very stable in extreme temperature conditions like 37°C during 1 month.

Taking into account all these results, we can speculate about the robustness of the primer pair and the plasmid for the standard curve since we consider that the incubation at 37°C during 1 month is an extreme condition that normally could not occur.

These results support the use of both materials generated in house (primer pair and plasmid for standard curve) as two very stable components of the low-cost qPCR system published by Aguiar et al 2014,^6^ and also support at the end the fact that this new method for hepatitis B VL quantification can be employed as an additional inexpensive technology to monitor the evolution of the hepatitis B disease of chronic patients involved in clinical trials, principally from the third world population, because with the adhesion of these two very stable components in the kit, we can contribute to minimize the expenses of this necessary test that, otherwise, will be prohibitive for people with economic limitations.
